# The Influence of Ambient Weather Conditions on Stated Preferences for Ecosystem Services Management

**DOI:** 10.1007/s00267-023-01839-4

**Published:** 2023-06-01

**Authors:** Sandra Notaro, Gianluca Grilli

**Affiliations:** 1 Department of Economics and Management, University of Trento, Via Inama 5/I, 38122 Trento, Italy; 2 Department of Land, Environment, Agriculture and Forestry, University of Padova, Via dell’Università 16, Legnaro, Italy; 3 Economic and Social Research Institute, Sir John Rogerson’s Quay, Dublin, Ireland; 4 Trinity College Dublin, Dublin, Ireland

**Keywords:** Ecosystem services conservation, Willingness to pay, Discrete Choice Experiments, Context-dependence, Sample selection bias, Generalized mixed logit model

## Abstract

One of the assumptions in stated preference studies is the stability of respondents’ preferences. This assumption might be violated in situations of context dependence, i.e., when the contingent situation influences respondents’ choices. Ambient weather conditions (AWCs) are one element of the context that may influence stated preferences. The literature suggests that AWCs affect people’s emotions, behaviors, and decision-making processes; however, the potential AWCs impact in environmental preference studies has not yet been investigated. This aspect is of high importance because context-dependent choices return biased willingness to pay estimates and affect the subsequent welfare analysis that informs public policy. To shed light on this important aspect of non-market valuation studies, we explore the effect of AWCs on preferences elicited with a Discrete Choice Experiment for ecosystem services management of a Nature Park. Results of a generalized mixed logit model evidenced a significant effect of AWCs on respondents’ choices, with good weather conditions leading to higher preferences and willingness to pay for ecosystem services management. This result, which is consistent with previous psychological studies, raises the issue of sampling design and reveals the importance of a sensitivity analysis of WTP. As this issue is still unexplored in stated preference studies, we also encourage undertaking similar studies to add a priori knowledge for more accurate *ex-post* calibration of WTP estimates.

## Introduction

The assessment of ecosystem services (ES) has become crucial for effective integration between conservation and development policies (TEEB 2010; Souliotis and Voulvoulis 2021). The economic value of ES is included in Cost-Benefit Analysis to quantify the alteration of ecosystem services (Wainger and Mazzotta 2011) and integrates conventional accounting at local, national, and regional levels (Costanza et al. (2014); 2017; Vargas et al. (2019); Cavalletti and Corsi 2021). The economic and social valuation of ES makes clear their benefits for society and thus can inform decision-making about specific land use developments (Rijal et al. 2021; Civeira et al. 2020; Cheng et al. 2019) that have an impact on different stakeholders and groups (Suwarno et al. (2016); Janssens de Bisthoven et al. (2022); Mulatu et al. 2022). Stated preference methods allow for monetary evaluate ecosystem services that are not exchanged in markets, and they can estimate both their use and non-use values (Bernués et al. 2019). According to Lienhoop and Schröter-Schlaack (2018) stated preference valuation thus contributes to the debate on the importance of ES, includes non-market ecosystem services in the decision, contemplates the distributive effect of ES management, and leads to better policy design. Among stated preference techniques, Discrete Choice Experiments (DCE) allow the economic valuation of each ecosystem service and their different levels of provision using the same metric, acknowledging explicit trade-offs in decision-making (Roesch-McNally and Rabotyagov, 2016; van Zanten et al. 2016). Moreover, DCE can provide more robust primary data on ES than quick valuation strategies for natural capital accounting (Grilli et al. 2021).

Stated preference methods assume that people have stable and consistent preferences and make rational choices (Hanley and Barbier 2009). However, behavioral anomalies are common in actual and hypothetical markets, leading to choices inconsistent with neoclassical microeconomics (Johnston et al. 2017). Some elements of the valuation context may boost such behavioral anomalies. Ambient weather conditions (AWCs) can be one of the elements of the valuation context affecting people’s decision-making processes and therefore stated choices, particularly in field surveys. In the presence of context dependence, welfare measurements might be biased and their interpretation difficult, leading to communicating incorrect policy recommendations to decision-makers. For this reason, the potential influence of AWCs on stated preferences and welfare estimates should be explored to understand potential biases arising in field surveys, such as sample selection bias.

The literature suggests that AWCs impact people’s life satisfaction (Schwarz and Clore 1983) and decision-making (Hirshleifer and Shumway 2003, Kamstra et al. 2003) through the mediating role of incidental emotions, which means that AWCs influence individuals’ emotions. In particular, psychological, financial, and economic studies showed that sunny weather makes individuals feel happy, leading to more actions and taking more riskier choices (Schmittmann et al. 2014). In contrast, during bad weather conditions, like windy days, people tend to choose the safest alternative, e.g., voting for the status quo in referenda (Bassi 2013). Good weather has also been proven to affect consumer spending positively (Murray et al. 2010).

A few stated preferences studies show the effect of incidental emotions on the formation of preferences (Notaro et al. 2019; Hanley et al. 2017; Araña and León 2008; Araña and León 2009). However, none of these studies examined the role of AWCs as a potential observable source of incidental emotions.

To fill this gap, in this paper, we investigate the effect of AWCs at the time of the survey on stated preferences for the conservation of ecosystem services, elicited with a Discrete Choice Experiment. If stated choices were affected by AWCs, the resulting welfare estimates are biased, and the resulting policies involving the protection and management of ecosystem services may not correctly represent individual preferences. The case study was Monte Baldo Local Nature Park (MBLNP), a Nature Park located in North-East Italy. The survey was administered to park visitors, and the ecosystem services evaluated were: maintenance of a diversity of plants and animals, local food provision, and recreation.

This study is grounded on the psychological and financial literature, which indicates that individuals are more likely to take action and select risky options on sunny days, and tests whether similar behaviors hold in the field of ecosystem service management. Two main hypotheses are investigated. The first hypothesis is that respondents would select costly scenarios compared to the status quo on sunny days and local management actions to conserve ES. The second hypothesis is that the status quo would be selected more frequently on windy days while local management actions would be less frequently selected.

The rest of the paper is organized as follows. The literature review is provided in the second section. In the third section, the DCE methodology and econometric modeling are presented. The fourth section includes results, while the fifth discusses the findings of weather effects on decision-making. Lastly, section number six offers some conclusions.

## Literature Review

AWCs are generally defined as the atmospheric conditions of a place at a given time. It is determined by the combination of atmospheric parameters such as wind, temperature, humidity, atmospheric pressure, sky cover, and precipitation (EPA).

Behavioral science and psychology’s insights suggest that AWCs influence choices and behavior. This effect is mediated by incidental emotions (Cohn 1990; Page et al. 2007) - “feelings at the time of decision not normatively relevant for deciding” (Lerner et al. 2015, p. 803). Incidental emotions influence cognitive processes, changing people’s choices (Rick and Loewenstein 2008; Blanchette and Richards 2010). It happens because preferences are not stable but are influenced by aspects of the environment in which a response is elicited (Slovic 1995).

The extant literature has shown three different but related effects of AWCs: 1) on human emotions, 2) on behavior, and 3) on the decision-making processes of people. A positive correlation between weather conditions (in terms of temperature, humidity, sunshine hours, and barometric pressure) and emotions has been shown by several studies (Goldstein 1972; Cunningham 1979; Sanders and Brizzolara 1982; Persinger and Levesque 1983; Howarth and Hoffman 1984; Parrott and Sabini 1990; Keller et al. 2005). Schwarz and Clore (1983) found that individuals are happier and more satisfied on sunny days. The weather-emotion relationship has also been studied by analyzing the content of tweets (Hannak et al. 2012). Results showed that aggregate sentiment follows climate, temporal, and seasonal patterns. On the other hand, Watson (2000) and Bauer et al. (2009) have not found any emotion-related climatic variable. Another part of the literature has demonstrated that weather influences behavior by impacting individual emotions. Altruism, for instance, is influenced by AWCs; that is, people are more willing to help others on sunny days than on cloudy days (Cunningham 1979; Guéguen and Lamy 2013). Finally, weather conditions have been shown to influence voting (Meier et al. 2016). On sunny days voters tend to prefer the riskiest candidate, while on cloudy and rainy days, the safest ones (Bassi 2013) or the status quo in referenda – i.e., the less risky alternative.

According to Schwarz (2012), sunny days, by making people happier, change information processing and influence people’s judgments and decision-making. The stock market is one area in which the influence of weather on choices has developed more (Bassi et al. 2013). Weather is likely to affect stock returns by influencing the emotions of the market participants. Sunny days are associated with positive emotions and higher risk-taking, as they are significantly correlated with equity returns and vice versa in rainy conditions (Saunders 1993; Hirshleifer and Shumway 2003; Chang et al. 2008; Lu and Chou 2012). Extreme conditions (temperature and humidity) have been proven to lower stock returns (Chang et al. 2006; Keef and Roush 2007; Yoon and Kang 2009; Kang et al. 2010), whereas high pressure induces positive emotions and higher equity returns (Schneider 2014). Other empirical studies have not found correlations between weather and stock returns (Krämer and Runde 1997; Trombley 1997; Pardo and Valor 2003). Keef and Roush (2002) obtained mixed results; they observed that temperature has a negligible impact on stock markets, whereas wind significantly negatively impacts stock returns.

Bassi et al. (2013) and Schmittmann et al. (2014) explained the relationship between weather conditions and market returns through the change in risk aversion. On good weather days, subjects are less risk-averse and take riskier behaviors, while during bad weather days, they make less risky choices being more risk-averse. Cao and Wei (2005) and Floros (2011) explained the different risk attitudes in stock markets through the aggressiveness or apathy of investors. Low temperature causes aggressiveness in investors, that are therefore more risk-taking, while high temperature causes apathy, so investors are more risk-averse, and equity returns are lower. Kamstra et al. (2003) and Dowling and Lucey (2008) found evidence that seasonal affective disorder (SAD) influences the risk attitude of stock investors and, consequently, stock returns.

Finally, good weather has been proven to affect consumer spending positively (Murray et al. 2010), and weather conditions have been associated with greater variety-seeking behavior in supermarkets (Tian et al. 2018).

Overall, most of the extant literature in psychological and economic areas has shown that weather factors affect emotions and, thus, behavior and decision-making processes of people, with good weather conditions leading to happiness and action and bad conditions to less action or no action. However, the effect of weather conditions on stated preferences for environmental conservation has not yet been investigated. This aspect is of high importance because weather conditions may affect choices, return biased willingness to pay estimates and affect the subsequent welfare analysis that informs public policy. Drawing on the two strands of research in psychology and finance, this study analyzes the impact of weather conditions on preferences elicited with a Discrete Choice Experiment for ecosystem services management of a Nature Park.

## Materials and Methods

### The Study Area

Data for this study was derived from a DCE survey aimed at evaluating visitors’ preferences for management measures of a Nature Park, i.e., the Monte Baldo Local Nature Park (MBLNP), which is situated in the Italian Prealps in the Autonomous Province of Trento.

Rising from Garda Lake, MBLNP covers 46.5 km^2^ from a few hundred meters above sea level to 2000 m. Nine protected areas are included in the Park, five Natura 2000 areas, and four regional or local nature reserves. The Park has particular importance for the biodiversity of vegetation due to the large quantity of species representative of diversified climatic varieties, from the Mediterranean to the Arctic Alpine ones. There are 28.7 floristic species for km², compared to 2.3 of other protected areas in the Province of Trento, including ten species protected by the European Union and 60 wild orchids. Thanks to this extraordinary flora biodiversity, it has been a popular destination for naturalists and pharmacists since 1400, so it was called “Hortus Italiae” (Garden of Italy). The Park is also important for fauna biodiversity. Numerous mammals, birds, reptiles, and amphibians live in the Park, including the rare Yellow-bellied Toad (*Bombina Variegata*). The Yellow-bellied Toad is a small aquatic toad with a colored belly living in central and south-eastern Europe. It is an upland species that prefers foothills and mountain regions and lives in different wetlands, like lakes and rivers, ponds, swamps, puddles, and stream pools. The Yellow-bellied Toad reproduces in continuous heavy rainfall, generally in non-shaded temporary pools with low or modest aquatic vegetation in or close to a forest. The European Union protects the Yellow-bellied Toad as many populations in Europe have disappeared or declined due to loss of suitable habitats, loss of connectivity between populations, drainage or water abstraction that makes the breeding ponds disappear, works on the forest during the time of reproduction, abandonment of pastures with consequent abandonment of the ponds for the watering of livestock, and eutrophication or pollution of habitats. For these reasons, the toad needs specific conservation measures to avoid extinction in Europe. These measures concern maintaining or restoring breeding ponds and dispersal corridors together with sites suitable for hibernation, maintaining cattle grazing, avoiding eutrophication and pollution of breeding ponds, and preventing forestry work during the breeding season (Council Directive 92/43/EEC– Annex II and IV). Considering the specific situation of Monte Baldo, the renovation and preservation of mountain puddles is the primary measure to be adopted to protect the toad by the Park. Due to its importance in biodiversity, Monte Baldo will be a candidate for the UNESCO World Heritage List.

The Monte Baldo Local Nature Park is included in the Reserves Network (RN), set up in the Province of Trento by a European Project funded under the LIFE program. The RN represents a more efficient way of managing ecosystem services in Natura 2000 areas and other regional or local nature reserves outside national and regional parks and in interconnection zones linking these protected areas. Therefore, the RN expresses the concept of an ecological network. This new way of managing protected areas is based on a bottom-up approach. The participation of local stakeholders, responsible subsidiarity, and the integration between conservation policies and sustainable local development is the fundamental principles on which the RN is based. Therefore, the areas included in the Reserves Network adopt management measures to protect biodiversity and develop sustainable traditional local economic activities, such as agriculture, crafts, and tourism, through a participatory approach (Martini et al. 2017).

Inside this framework, in 2008, the Brentonico Reserve Network was established. It was the first RN activated by the Province of Trento. It became Monte Baldo Local Nature Park in 2013 because it reached specific naturalistic and territorial criteria required by the Low to be called a Local Nature Park. Local stakeholders’ participation is crucial at MBLNP for implementing an integrated management system in which the preservation of flora and fauna biodiversity interacts with agriculture and tourism to implement socio-economic development projects respectful of the natural environment. A critical goal of the Park is safeguarding and promoting traditional activities, specifically agricultural activities, for the benefit of sustainable tourism. Locally produced food benefits significantly from the presence of different species of herbs and flowers, such as some typical cheeses produced in mountain pastures and sold to tourists in the summer. The Park attracts tourists who visit it for walks, trekking, mountain biking, picnics, and to enjoy the landscape, especially during the summer season. Thanks to the mild climate, it offers visitors countless possibilities for naturalistic excursions from spring to late autumn.

Before the establishment of the RN, the Province of Trento centrally managed this area, and it was impossible to implement management measures strictly linked to the local environmental and socio-economic conditions.

### Survey Design and Administration

The evaluation of preferences and willingness to pay for the management and conservation of selected ecosystem services of the MBLNP was performed using a Discrete Choice Experiment (Louviere et al. 2000). The DCE is a survey-based method that aims at understanding respondents’ preferences for management scenarios. These management scenarios are described by some relevant management actions, which are combined and presented to the respondents iteratively in a series of choice situations. Respondents answer the questionnaire by selecting the preferred scenario in each choice situation.

Data were collected on-site with a face-to-face questionnaire to visitors of the Park from June to September 2017. Four interviewers asked every second tourist they met in different areas of the Park to participate in the survey to have a sample as random as possible.

The questionnaire was developed based on widely-accepted guidelines for DCE (Riera et al. 2012) and divided into four thematic sections. The first section included information about the management of the Park. The second section explained attributes and levels and contained the choice cards. To mitigate hypothetical bias, we included a policy consequentiality script (Carson and Groves 2007). The last two sections contained behavioral^1^ and socio-demographic questions.

Relevant attributes were tested with experts, naturalists, and managers of the RN and the Park. The initial set of candidate attributes resulted from a long participatory process, during which local stakeholders discussed the biodiversity conservation and development objectives to be achieved and the management measures to be implemented in the Park. Four attributes were selected considering their importance in local park management and their actual possibility to be implemented. Specialists and local stakeholders also defined the different management measures associated with the attribute levels. Attributes and attribute levels are available in Table 1.

**Table 1:** Attributes and levels used in the choice cards

Attributes	Description	Levels	No local management (centralized management) (CMan)
Biodiversity of the meadows (BioM, BioH)	Protection through sheep pasture and mowing of meadows	1. Low (no action for biodiversity protection)	Low biodiversity in the meadows, no action to protect it
		2. Medium (controlled sheep grazing)	
		3. High (mowing of meadows)	
Protection of the Yellow-bellied Toad (Toad)	Protection through the restoration and conservation of mountain puddles	1. No (no protection)	No protection
		2. Yes (action for its protection)	
Trails (Trails1, Trails2)	Restoration and improvement of the trails	1. No (no action)	No restoration or improvement
		2. Restoration (make trails safe and clean)	
		3. Restoration and improvement (make the paths safe and clean, put the signs on the paths, create topographic maps in paper and digital format)	
Local organic products (Prod)	Availability of local organic products in farms, alpine huts, markets, restaurants, and hotels	1. No (no local products)	No presence of local organic products
		2. Yes (there are local products)	
Entry ticket (Cost)	Cost of the day ticket to enter the park	3€, 6€, 9€, 12€, 15€, 18€	0 €

Two attributes were related to local management measures associated with supporting services, i.e., protection of biodiversity of the meadows through sheep pasture and mowing of meadows, and protection of the Yellow-bellied Toad through the restoration and conservation of mountain puddles; one to provisioning services, namely local organic food production, and one to cultural ecosystem services, specifically the restoration of trails for recreation.

The attributes of protection of the Yellow-bellied Toad and local organic food products had two levels of policy action, while biodiversity of the meadows and trails had both three levels. The payment vehicle for the monetary attribute was an entrance ticket to the Park, which is necessary for the local community to co-fund local management initiatives. Six price levels were used based on previous similar surveys conducted in neighboring areas (Gios et al. 2006; Notaro et al. 2006; Scarpa et al. 2011).

The status quo alternative was associated with centralized management by the Province of Trento. With central government, local stakeholder participation in management is no longer possible, as well as the implementation of the actions specifically designed for local conditions presented to respondents in the policy scenarios. Since the status quo does not include investments in the described local management measures, this alternative is cost-free, and all the attributes are presented at the lowest level (low biodiversity of meadows, no specific measures to protect the Yellow-Bellied Toad and to recover trails, and no availability of local organic products). The two policy alternatives presented measures identified by local stakeholders at different prices (see questionnaire in the Appendix).

During the survey, interviewers collected data on current weather conditions at the time of the interview (Table 2). Weather conditions were identified into five categories (sunny, partly sunny, mostly cloudy, cloudy, rainy), subsequently recoded on a two-level scale (good weather, which contains sunny, partly sunny, and bad weather, which contains mostly cloudy, cloudy, and rainy). Wind conditions were identified in three categories (calm day, breezy, and blustery). Individual self-reported weather conditions were also collected among the respondents to test the consistency of the weather conditions and their perception by the respondents.

**Table 2:** Weather conditions observed at the time of the survey and coding of the dummy variables

Weather conditions	Coding of the dummy variables
Sky	1 = sunny or partly sunny
	0 = mostly cloudy, cloudy, or rainy
Wind	1 = calm day
	0 = breezy, blustery

A pilot of 66 visitors was used to test attributes, attribute levels, and wording and to estimate priors for the experimental design. An Optimal Orthogonal Choice Design (Street and Burgess 2007; Rose and Bliemer 2009) was generated for the pilot, while a sequential design (Ferrini and Scarpa 2007; Bliemer et al. 2008) was prepared for the final survey. The priors obtained in the pilot informed the first D-efficient design and the parameters estimated with the first 383 questionnaires were employed to create a second, more efficient design. NGene (ChoiceMetrics (2014)) was used to generate the designs. The interviewees completed 12 choice cards.

The answer format was the Best-Worst (Flynn et al. 2007; Louviere et al. 2013). Compared to complete ranking methods, Best-Worst is easier to answer for respondents because best-worst alternatives are more easily identified (Scarpa et al. 2011).

### Econometric Model

Discrete choice experiments are based on the Random Utility Theory (RUT) (Manski 1977) and Lancaster’s Theory (1966) which states that the utility that a consumer obtains derives from each characteristic of the good. According to the RUT, the utility of respondent *n* for alternative *i* in the choice situation *t* can be represented with a linear in the parameters utility function:

1 $$U_{int} = \beta X_{int} + \varepsilon _{int}$$

where *β* are the parameters, *X*_*int*_ are the attributes of the alternative *i* and *ε* the error term. The probability of choosing an alternative can be calculated using the conditional logit model, which assumes preference homogeneity or more flexible models that account for heterogeneity in preferences (Train 2009; Mariel et al. 2021).

We analyzed the sequence of choices with the Generalized Mixed Logit (GMX) model (Fiebig et al. 2010; Hensher et al. 2015), a flexible model that accounts for taste and scale heterogeneity (Train 2009). This model assumes that coefficients are individual-specific and follow a random distribution, for which a location and a scale parameter are estimated:

2 $$P_{ni} = {\int} {\frac{{e^{\beta _n^\prime X_{:ni}}}}{{\mathop {\sum }\nolimits_j e^{\beta _n^\prime X_{ni}}}}\varphi } \left( {\beta {{{\mathrm{|}}}}b,\,\Omega } \right)d\beta$$

In which:

3 $$\beta _n = \sigma _n\left[ {\beta + {{\Delta }}z_n} \right] + \left[ {\gamma + \sigma _n\left( {1 - \gamma } \right)} \right]\Gamma v_n,$$

where:*σ*_*n*_ is the scale factor of the error term that varies among individuals. It is assumed to be log-normal distributed with mean $\bar \sigma$ and standard deviation *τ*, which is the coefficient of the unobserved scale heterogeneity. Γ*v*_*n*_, the residual preference heterogeneity, also varies with the scale. The variation is controlled by a weighting parameter *γ* that indicates how variance varies with scale. When *γ* = 1, only the mean parameters are scaled by an individual random factor, whereas when *γ* = 0 mean and taste heterogeneity parameters are scaled.

In our study, we used the Best-Worst question format, in which respondents reported the preferred and less preferred alternatives among the three alternatives presented in each choice card. Since with the Best-Worst format, we have two choice observations from each choice card, we estimated choice probabilities as the product of the probability of the best choice and that of the second-best (Luce and Suppes 1965; Scarpa et al. 2011):

5 $$P_{ni}\left[ {ranking\,i_1,i_2,i_3} \right] = {\int} {\frac{{e^{\beta _n^\prime X_{ni_1}}}}{{\mathop {\sum }\nolimits_{i = i_1,\,i_2,i_3} e^{\beta _n^\prime X_{nij}}}} \times \frac{{e^{\beta _n^\prime X_{ni_2}}}}{{\mathop {\sum }\nolimits_{i = i_1,\,i_2} e^{\beta _n^\prime X_{ni}}}}} \varphi \left( {\beta {{{\mathrm{|}}}}b,\,\Omega } \right)d\beta$$

To test if weather conditions lead to differences in observed choices, the attributes and the constant parameter were interacted with dummies representing being interviewed under different sky and wind conditions (Table 2). The cost was entered as a continuous variable. We considered the possibility that respondents’ choices are endogenous with respect to weather conditions. Endogeneity may arise when visitors decide to visit in a specific weather condition, which is unobservable and correlated to the error term. For instance, some visitors may decide to visit in good weather conditions to enjoy the surroundings, whereas other visitors may visit in bad weather conditions to increase the likelihood of being isolated. We believe that this source of endogeneity does not affect the results of our study because there is no link between weather conditions and the type of management of natural areas. This endogeneity potentially affects the number and frequency of visits but does not influence willingness to pay for the type of management of the natural area. Respondents would still visit the natural area in the weather conditions they prefer, regardless of the type of management.

WTPs were estimated using the Krinsky-Robb method (Krinsky and Robb 1986) with 10,000 draws.

## Results

Interviewers intercepted 819 visitors, and considering 24 observations per respondent, the final dataset was composed of a total of 19,656 observations. About half of the respondents were male (50.06%), and the average age was 43. Most respondents had a high school degree (53 percent), while the share of respondents with a university degree was around 37 percent. Forty-four percent of respondents have a net income between €10–30 thousand. The socio-demographic characteristics of the sample are comparable to that of the regional tourists (P.A.T. Provincia Autonoma di Trento (2016)).

Interviewers recorded weather conditions before each questionnaire administration. Out of 819 questionnaires, 610 were collected in good weather conditions (about 74%), while the remaining 209 (26%) were in bad weather conditions. The balance of respondents collected in good and bad weather is comparable to the annual weather variation in the region. In fact, in Trentino, the average number of annual rainy days is 88, corresponding to 24% of the year, while the remaining 76% is sunny or partly sunny. To allow comparisons between individual choices, these two samples should be similar in terms of socio-demographics and only differ by AWCs at the time of the survey. Therefore, statistical tests were conducted to assess whether the samples of respondents allocated in the two groups were similar in terms of their socio-demographics. These tests suggested that respondents in the good and bad weather samples were comparable in terms of gender, education, and income (Table 3). The test on respondents’ age returned a significant result, which indicates that the two samples differ by age. Despite this result, the average age is similar across groups (40 and 43 years in the bad and good weather samples, respectively) and should not significantly influence the results. Based on these tests, it was concluded that samples were similar enough to allow robust statistical analyses.

**Table 3:** Good and bad weather samples statistics

Socio-demographic characteristics	Mean of total sample	Mean of good weather sample	Mean of bad weather sample	*T*- statistic	*P* value
***T*-tests for numeric socio-demographics**					
Age	43.14	43.88	40.97	−2.57	0.010
Gender (female)	0.50	0.51	0.46	−1.28	0.201
**Mann-Whitney tests for categorical variables**					
Education	2.16	2.13	2.26	1.72	0.086
Income	1.99	2.02	1.91	−0.78	0.435

Education is coded 0 = Grade 10 or less; 1 = Technical School (3 years); 2 = High School Diploma; 3 = Undergraduate Degree (Bachelor’s); 4 = Graduate Degree (Master’s, Doctorate); Gender is coded 1 if female, 0 if male. Income is coded: 0 = less than 9.999; 1 = 10.000–19.999; 2 = 20.000–29.999; 3 = 30.000–39.999; 4 = 40.000–59.999; 5 = 60.000–79.999; 6 = 80.000–99.999; 7 = 100.000 or more

Another preliminary assessment was the impact of AWCs on self-reported individual emotions (Table 4). Concerning emotions, AWCs had a significant impact on self-reported happiness and boredom. Respondents interviewed in good weather conditions were more likely to be happy and less likely to be bored compared to respondents in the bad weather sample. Other emotions (anxiety, calmness, irritability, and sadness) were not affected in a statistically significant way. This result is in line with the literature on the impact of AWCs on emotional states.

**Table 4:** *T*-tests on emotions between good and bad weather samples

Emotion	Mean of bad weather sample	Mean of good weather sample	Diff	*T*-statistic	*P* value
Anxiety	1.54	1.46	−0.08	0.94	0.348
Boredom	1.8	1.54	−0.26	2.53	0.012
Calmness	5.49	5.62	0.13	−1.05	0.293
Happiness	4.64	4.88	0.24	−1.82	0.069
Irritability	1.55	1.58	0.03	−0.34	0.734
Sadness	1.5	1.41	−0.09	0.96	0.34

The last preliminary check related to the impact of AWCs on individual perceptions of the weather (Table 5). When asked to indicate on a 7-point scale the extent of satisfaction with the day’s weather conditions, respondents in the good weather sample expressed an average exceeding 6, while respondents in the bad weather sample of about 4. The averages were statistically different after a *t*-test on answer distributions between samples. This result indicates that respondents in the good weather sample are more likely to be satisfied with the weather conditions than those in the bad weather sample.

**Table 5:** *T*-test on perceived weather based on actual AWC at the time of the survey

	Mean of bad weather sample	Mean of good weather sample	Diff	*t*-statistic	*P* value
Perceived weather	4.8	6.23	1.43	−12.09	0.000

The preliminary analysis confirmed that AWCs significantly impacted emotions and perceived weather conditions. The following step investigates the impact of AWCs on ES management preferences as collected from the DCE. Table 6 shows the results of the Generalized Mixed Logit model.

**Table 6:** Results of the Generalized Mixed Logit Model

Attributes	Mean Coefficient	*t*-test	Standard dev. Coefficient	*t*-test	Odds ratio
BioM	0.282***	3.87	0.415***	5.48	1.33
BioH	0.757***	9.45	0.552***	7.41	2.13
Toad	1.036***	10.89	0.987***	14.81	2.82
Trail1	0.795***	8.48	0.545***	5.55	2.21
Trail2	1.461***	12.82	0.760***	7.66	4.31
Prod	0.976***	12.45	0.571***	10.07	2.65
CMan	−5.691***	−12.95	4.452***	16.25	0.003
Cost	−0.234***	−48.12			0.79
BioM Sky	0.007	0.08	0.307***	2.84	1.01
BioH Sky	0.028	0.31	0.177	1.24	1.03
Toad Sky	0.237**	2.36	0.269**	2.19	1.27
Trail1 Sky	0.091	0.86	0.456***	3.70	1.10
Trail2 Sky	0.042	0.35	0.855***	7.79	1.04
Prod Sky	0.136*	1.70	0.363***	5.24	1.15
BioM Wind	0.050	0.33	0.032	0.06	1.05
BioH Wind	0.187	1.16	0.346	1.19	1.21
Toad Wind	0.414**	2.22	0.303	0.71	1.51
Trail1 Wind	0.143	0.71	0.592***	2.72	1.15
Trail2 Wind	0.309	1.33	0.433	0.91	1.36
Prod Wind	0.074	0.51	0.009	0.02	1.08
CMan Sky	−0.980**	−2.35	2.793***	8.14	0.38
CMan Wind	−2.440**	−2.45	2.277	1.60	0.09
TauScale	1.017***	27.07			
GammaMXL	0.144***	6.57			
Sigma	0.948	0.89			
Obs	19656				
Respondents	819				
log_L	−10954.04				
McFadden’s R^2^	0.493				
AIC	1.119				
BIC	1.137				

** and *** indicate significance levels at 5% and 1%, respectively

The value of McFadden’s R^2^ indicates that the model fits the data well. All main-effects parameters have the expected signs and are statistically significant at a 1% level. The cost coefficient is negative, suggesting diminishing marginal utility with higher prices. The coefficient for centralized management (CMa) is negative, indicating that respondents prefer local management measures of ecosystem services. Indeed, non-monetary attributes are all positive, indicating positive preferences for the described local management measures of ES in the Park. Consistent with the economic theory, the three-level attributes show higher coefficient values for higher levels of management’s output. Overall, visitors indicate that they favor both just the restoration and the restoration and enhancement of trails. However, they prefer additional services (enhancement), such as signs along the trails, topographic maps, and digital trail maps. Protecting the Yellow-bellied Toad is the second preferred management measure, and incentivizing local organic products is the third. Flora biodiversity is associated with the smallest contribution to the utility function of visitors since the two associated parameters are the smallest in absolute terms. Visitors prefer actions that lead to obtaining a high level of biodiversity—mowing meadows in autumn—compared to actions that allow obtaining a medium level, namely sheep grazing. Table 6 contains a column where odds ratios are displayed. Odds ratios indicate the odds of choosing an alternative that contains a particular attribute compared to alternatives without that attribute. In terms of odds ratio, alternatives including restoration and improvement of trails were 4.3 more likely to be chosen with respect to alternatives with no interventions on trails. The restoration-only intervention was 2.21 times more likely to be selected compared to the reference level of no interventions. Other high odds ratios exceeding 2 were associated with protecting Yellow-bellied toads (2.82) and maintaining a high level of biodiversity (2.13).

The two interaction terms CMan Sky and CMan Wind, are negative and significant at a 5% level. This means the likelihood of choosing centralized management decreases during sunny and calm days. This indicates that good weather conditions increase the utility of local management of ES and related policy interventions.

The second panel of Table 6 displays interaction terms between attributes and the variables of interest. As a general interpretation rule, significant interaction coefficients are reflected in statistically significant effects of the weather on preferences and WTP for that specific attribute. This analysis suggests that weather had little influence on single attributes. The interactions coefficients Toad Sky and Toad Wind are positive and significant at a 5% level, indicating that respondents show higher preferences for protecting the Yellow-Bellied Toad during a sunny and calm day. A sunny day also positively influences preferences for local organic food products, being the related coefficients positive and significant at a 10% level. In general, the impact of AWCs on single attributes is small, probably because the experiment was designed to explore preferences for local versus central management of the Park. Therefore, ACWs impact is reflected in the status quo coefficients, which capture the overall preference rather than on single attributes.

The highly significant standard deviations of the main effect coefficients demonstrate preference heterogeneity in our sample. It is worth noting that whereas heterogeneity among respondents is identified for sky conditions, no heterogeneity is found for wind conditions. The positive influence of a calm day on preferences for local management measures of ES does not change among respondents.

Scale heterogeneity is also present, as the scale coefficient (TauScale) and the weighting parameter (GammaMXL) are both positive and highly significant. The value of the weighting parameter, which is close to zero, indicates a high dependence of residual taste heterogeneity from the scale factor.

Observing WTPs (Table 7), all of them are positive except that one for the central management. The highest willingness to pay is related to the improvement of trails. Specifically, tourists were willing to pay roughly twice as much for a significant improvement in walking paths than a medium improvement. Protecting the Yellow-bellied Toad was the second measure valued positively, followed by the availability of local food products. The lowest willingness to pay is given to flora biodiversity.

**Table 7:** WTPs with different weather conditions (in €)

	Weather conditions		
Attributes	Mostly cloudy, cloudy, or rainy	Sunny or partly sunny	Wind: a calm day
BioM	1.208***	1.215***	1.259***
BioH	3.239***	3.267***	3.426***
Toad	4.433***	4.670***	4.846***
Trail1	3.401***	3.491***	3.543***
Trail2	6.250***	6.292***	6.559***
Prod	4.176***	4.312***	4.250***
CMan	−24.347***	−25.327***	−26.788***

*** indicate significance level 1%

The negative WTP for the central management indicates that respondents would be worse off if they had to give up management measures of ES, which can be implemented if local communities manage MBLNP, so they should be compensated.

WTPs for the central management that account for weather effect are larger in absolute values on sunny and calm days. This means that during days with good weather conditions, people ask for higher compensation to give up the opportunity to have the offered local management measures of ecosystem services. Furthermore, WTPs for the different management measures to conserve ES turn out to be higher during sunny and calm days. WTP values are statistically different between the good weather and bad weather samples in correspondence of statistically significant interaction coefficients, namely the conservation of the Yellow-bellied Toad (WTP for the toad is larger on sunny days and in the absence of wind) and the availability of local organic products (WTP is larger in sunny days). The most relevant difference between the two samples is associated with the status quo variable, which captures preferences for central management. Overall, tourists prefer local management and want to be compensated if management becomes centralized. On average, respondents stated to be willing to accept €1 more as compensation on sunny days and €2 more on non-windy days.

## Discussion

Our DCE evidenced positive preferences and WTPs for all local management measures of the proposed ecosystem services, indicating that visitors favor the RN as a management tool to provide ES. The negative coefficient associated with central governance indicated that, on average, visitors more likely prefer scenarios with increased local management of ES. Concerning the research hypothesis of this paper, we reported a significant impact of AWCs on preferences, which was expressed in the interaction coefficients of every single management measure and the central management. On the first hypothesis, sunny days were associated with an increased likelihood of selecting local management policy alternatives over centralized management and alternatives that included the protection of the Yellow-Bellied Toad and the availability of local organic products. On the second hypothesis, calm days were also associated with an increased probability of selecting local management policy alternatives and the protection of the Yellow-Bellied Toad. In the case study, policy alternatives represent changes in ecosystem services management that come at a cost for the respondents. In contrast, centralized management denotes the “no action” alternative, which is provided at no cost to respondents. This effect translates into negative WTP for central management, meaning that higher compensations are required to give up the opportunity to have the local policies applied in the Nature Park to provide ES. Moreover, respondents show higher WTPs for locally offered management policies during sunny and calm days. Overall, these results are in line with those obtained by Murray et al. (2010), that found a positive relationship between good weather and consumer spending, and the vast psychological and financial literature on the effect of good and bad AWCs on behavior and decision-making processes of people, with good weather conditions leading more to some action and bad conditions to less or no action.

This study contributes to the general literature on individual preference stability. While cost-benefit analysis assumes that preferences are stable and independent from the choice context, there is evidence that this might not be true in all situations. The impact of AWCs is likely to be revealed by the mediating effect of emotions (Cohn 1990, Page et al. 2007); therefore, different AWCs cause different individual emotions. The available contributions on the impact of emotions on stated preferences (Araña and León 2008; Araña and León 2009; Hanley et al. 2017; Notaro et al. 2019) show mixed results. However, none of these studies explicitly investigates factors influencing emotions. The types of emotions, the factors influencing them, and choice contexts are manifold, and decisions may be affected (or maybe not) in several ways. More research is necessary to draw conclusions.

There is no obvious solution to address context dependence and its biases. However, some good practices that reduce the risk of incorrect policy recommendations based on welfare estimates biased by AWCs may be identified. The first good practice is to record weather conditions during on-site data collection. Controlling for AWCs during data analysis may help reduce AWCs biases. Supplementing results with information on weather conditions and other context-dependent information helps policymakers rule out study biases or understand the study’s weaknesses. Providing policymakers with a sensitivity analysis of results to changes in weather conditions is therefore highly recommended. Another good practice is to consider weather-related sampling selection in DCE studies. When collecting data on-site, it is common practice to interview respondents during good weather days because it is more likely to meet visitors, and the data collection is easier. This practice, however, may return over-estimated WTPs; therefore, a systematic balanced sample selection process design that accounts for data collection in different weather conditions would be recommended. However, this solution is not often practical because meeting visitors on bad weather days is hard, and sample collection may be severely delayed. An important question is, therefore, how and how much WTP estimates and welfare analysis are likely to be biased by AWCs. This study found that good AWCs resulted in lower preferences for the status quo (the central management) and larger WTPs for alternative policy scenarios regarding ES management, and bad AWCs led to opposite results. Therefore, the impact of good weather conditions is likely to cause an upward bias to WTP estimates and a higher welfare effect of a new policy and vice-versa. Since field surveys are usually conducted in good weather conditions, an upward bias typically prevails. An upward bias is probably not important when benefits largely exceed costs. Weather effects need deeper attention when the output of a policy is uncertain, and costs may exceed the benefits. The output of a policy cannot be predicted during the phase of sample collection; however, the literature on similar studies may provide some important a priori knowledge and indications to be used for ex-post calibration of WTP estimates. A major limitation of this study is that it cannot alone give a calibration scale for WTP estimates to account for weather condition effects. Further studies to bring additional knowledge on the extent of the effect of AWCs on welfare estimates are therefore necessary. Once a basis to calibrate responses with AWCs is available, it will be possible to keep track of AWCs and statistically account for potential weather effects when aggregating data.

One last aspect to consider is that individual perceptions about the weather might be influenced by weather conditions in respondents’ place of residence. This study surveyed only Italian tourists, 95% from Northern Italy; therefore, the impact of weather conditions in respondents’ hometowns is likely to be small because the weather is similar in Northern Italy. However, perception might be considerably heterogeneous when respondents are international or from locations far from the study area. For this reason, the questionnaire should always capture individual perceptions about the weather to control if actual weather conditions disproportionately differ from individual weather perceptions.

## Conclusions

The importance of ecosystem services has been acknowledged in recent decades, but they still remain marginal in policy decision-making (Costanza et al. 2017). The economic valuation of benefits deriving to society from ES is useful for their recognition in public policy, creating an incentive to preserve the natural capital for the current and future generations. Moreover, many policy decisions involve trade-offs between various ES, and valuation can highlight the relative importance of each ES. In this light, Discrete Choice Experiments allow to economically evidence the trade-offs among different ecosystem services (van Zanten et al. 2016). However, some elements of the valuation context may influence willingness to pay, leading to biased welfare estimates and wrong policy recommendations. Weather conditions at the time of the survey can be one of these contextual factors.

Welfare-based economic policy is grounded on the assumption of rationality of the individuals and preference stability. The psychological literature and behavioral research have long demonstrated that preferences are not stable but are influenced by aspects of the environment in which a response is elicited (Slovic 1995; Fischer and Genk 2011). Specifically, the above-mentioned behavioral literature suggests that AWCs have an impact on individuals’ rationality and affect choices through the mediated role of incidental emotions. This evidence is obtained in the context of real choices and behaviors; however, it is reasonable to speculate that AWCs will also affect stated choices. This aspect is of high importance in non-market valuation because AWCs at the time of the interview may represent an element of context-dependence for the assessment of individual choices, which violates the hypothesis of preference stability and biases willingness to pay estimates. Hence incorrect information would be communicated to policymakers. Policy advice based on the assumption that people make rational choices has approached its limits, given the evidence for the influence of behavioral factors in decision-making (Menzel 2013).

Our study tested the impact of external weather conditions on stated preferences and WTP for management measures of ecosystem services in a Nature Park. The Generalized Mixed Logit model evidenced a significant effect of the weather variables on preferences consistent with psychological and financial literature findings. Good weather conditions were associated with higher preferences for local management measures of ES, whereas bad weather conditions with higher preferences for centralized management, which represents the “no actions” alternative.

The influence of AWCs on respondents’ choices in stated preferences field surveys raises the issue of sampling selection because field surveys are likely to occur in good weather conditions. To avoid communicating incorrect policy recommendations to decision-makers, a systematic balanced sample selection process design should be employed, in which interviews are balanced over different weather conditions. Since this solution is not often feasible, we suggest further studies on this topic to identify scale calibration factors that allow, after keeping track of AWCs, to account for potential weather effects when aggregating data statistically. Providing policymakers with a sensitivity analysis of results to weather conditions and other context-dependent information is also recommended since it would allow policymakers to understand the robustness of the study results.

The effect of weather conditions on respondents’ choices can be thought to be more evident in field surveys. However, the financial literature demonstrates that this bias exists in stock exchanges, even if buyers and sellers trade inside. Online surveys are gaining popularity in academic research. People typically answer an online survey indoors, but financial studies evidence suggests that ambient weather conditions may also influence stated preferences in online surveys. This paper advocates the need for further inquiry into this important aspect.

### Supplementary Information

Supplementary Information

## Footnotes

1 The effect of the Theory of Planned Behavior on preferences is examined by Grilli and Notaro (2019).
