# Supplementary material for: The Influence of Ambient Weather Conditions on Stated Preferences for Ecosystem Services Management
Source: Environ Manage. 2023 Jun 1;72(6):1228–40. doi: 10.1007/s00267-023-01839-4 (PMC10570176; doi:10.1007/s00267-023-01839-4)
Supplement: Supplementary file 1 — Supplementary Information [file 267_2023_1839_MOESM1_ESM.docx]

Appendix – The questionnaire


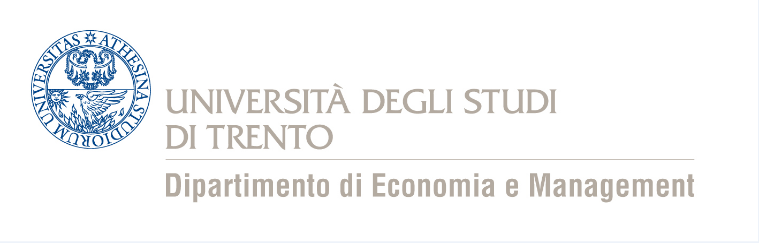

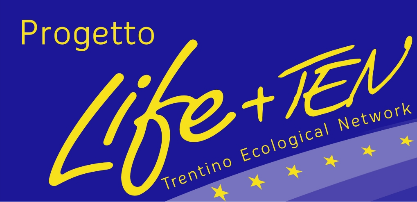


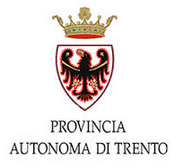


SURVEY

**MANAGEMENT OF MONTE BALDO LOCAL NATURAL PARK**

This survey collects tourist preferences for alternative management strategies of the Monte Baldo Local Natural Park. The study is framed within the action D2 of the European Union-funded project LIFE11/NAT/IT/000187 "TEN" - Trentino Ecological Network."

*The interview will comply with your privacy rights prescribed by the Italian Decreto Legislativo 196/03, articles 13 and 22 comma 2, which regulates data protection policies.*

## The questionnaire will take approximately 15 minutes.

***We thank you for your precious collaboration*.**

**Some questions to start**

**1. Do you practice outdoor activities regularly (mountain walks or hiking, climbing, mountain bike, nordic walking, etc.?)**

 No

 Yes, occasionally

 Yes, frequently

2. Is it your first visit to Monte Baldo? YES  NO 

**3. Are you on a daily trip?**  YES  NO 

4. If you are on a multi-day holiday, how many days will you spend in Monte Baldo? _____

**Some information before continuing**

The Network of Natural Reserves of Brentonico (Rete di Riserve di Brentonico) was established in 2008 and transformed into Monte Baldo Natural Park in 2013. The Reserve Network was established in Trentino to manage Natura 2000 areas more effectively and according to the EU legislation.

| In a Reserve Network:   - The **population is engaged and participates** in the decisions related to the management of natural areas and the socio-economic development of the territory. - The **local socio-economic development policies are integrated with environmental conservation.** | 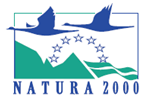 |
| --- | --- |

**This approach is currently used to manage Monte Baldo Local Natural Park.**

| The Park is rich in **FLORA BIODIVERSITY.**  In particular, there are:   - **28,7 flower species per km^2^** compared to 2,3 species per Km2 of other regional parks. - **138** endangered flower species in the region. - **10** EU-protected species. - **60** wild *orchids*. | 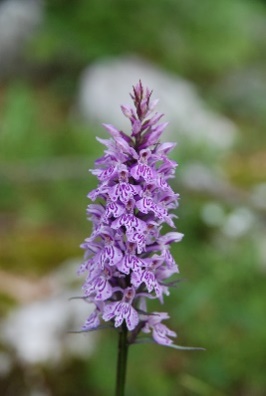 |
| --- | --- |
| The Park is rich in fauna biodiversity. There are numerous mammals, birds, reptiles, and amphibians, even rare ones, such as:   - **YELLOW-BELLIED TOAD**, a small toad strictly protected by the European Union, which requires specific conservation interventions to avoid extinction. | 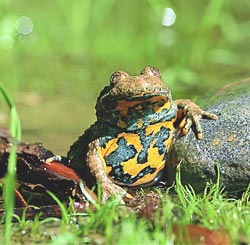 |

**Management actions of Monte Baldo Local Park to protect biodiversity.**

**To protect flower and meadow biodiversity**, including orchids:

- - Mowing
  - *Controlled sheep grazing*

Sheep grazing is less effective for the local biodiversity than mowing because sheep manure makes the meadows less favorable to orchid growing.

- With mowing, it is, therefore, possible to reach a high level of biodiversity,
- With sheep grazing, a medium level of biodiversity.

- Actions to protect the **Yellow-bellied toad:**
- Protect alpine pools where the Yellow-bellied toad live
- Restore alpine pools that disappeared in the last few years.


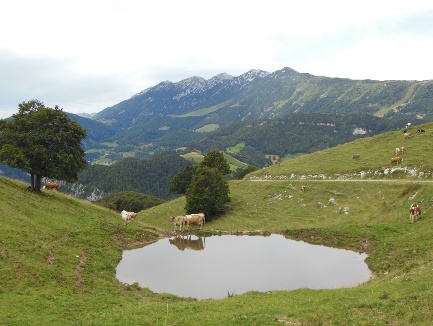


**These actions have been promoted by the local population and funded by Monte Baldo Natural Park.**

**Management actions of Monte Baldo Park for a sustainable tourism**

Other management actions concern the development of sustainable tourism, namely:

- **Mountain trails**

| - Restore trails - Restore and improvement trails.   Trails improvement might be achieved by placing signals, topographical maps, digital maps, and pictures on the website. | **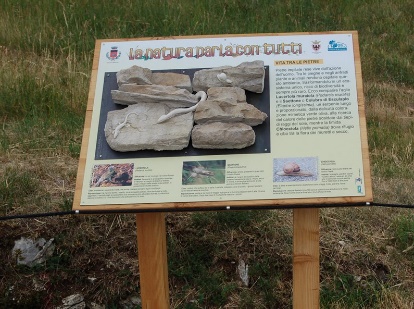** |
| --- | --- |

- **Locally supplied organic products.**

| Encourage and improve the availability of local products in farms, seasonal markets, alpine pastures, restaurants, and hotels. | 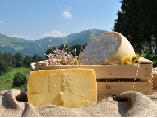 |
| --- | --- |

**These actions have been promoted by the local population and funded by Monte Baldo natural park.**

**The current park management is carried out with projects proposed by the local population.**

**We now ask you to consider some alternative park management**

Each alternative includes:

1. The level of meadow **Biodiversity** (*high, medium, low*)
2. The **protection of the Yellow-bellied** toad through the restoration and conservation of mountain pasture pools for (*yes or no*)
3. Restoration and enhancement of trails (*restoration and enhancement, only restoration, no restoration or enhancement*)
4. The availability of **local organic products** in farms, mountain huts, markets, restaurants, and hotels (*yes or no*)
5. The cost of the entry ticket to the Park

This is an example of a card with 3 scenarios.

**The third scenario is a central management scenario, in which the local population doesn’t have a role in the Park management.**

| **Card 0 - example** | | **Scenario A** | **Scenario B** | **No local management** |
| --- | --- | --- | --- | --- |
| 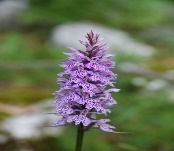 | **Meadow biodiversity** | Medium | High | Low |
| 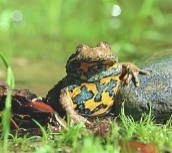 | **Yellow-bellied toad protection** | No | Yes | No |
| **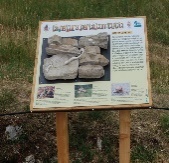** | **Trails** | Restoration | Restoration | No |
| 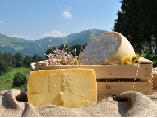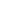 | **Local organic products** | No | Yes | No |
| 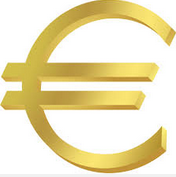 | **Entrance ticket** | 8 € | 12 € | 0 € |
| Which is your **preferred** scenario? | |  |  |  |
| Which one of the remaining is your **least preferred** scenario? | |  |  |  |

**How to fill out the cards**

We will now show you 12 cards.

**Each card contains 3 different scenarios.**


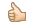
In each card, please select:

**(1)** your **preferred** scenario among the 3

**(2)** your **least preferred** scenario among the 2 remaining scenarios

***Important! Results will be presented to the local decision-makers and population to improve Park management.***

*For this reason, it is important that your answers are as close as possible to your preferences so that better decisions can be made.*

**
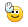
 We encourage you to consider all alternatives carefully and the associated cost.**

**Imagine that a ticket should be paid for now.**

**Answer the cards considering the cost for you only, without adding up the ticket your family or friends with you should pay.**

**There are no right or wrong answers; we are only interested in your preferences!**

| **Card 1** | | **Scenario A** | **Scenario B** | **No local management** |
| --- | --- | --- | --- | --- |
| 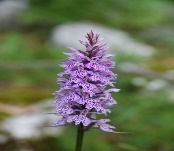 | **Meadow biodiversity** | Low | Medium | Low |
| 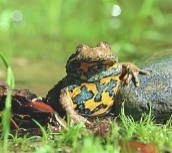 | **Yellow-bellied toad protection** | No | Yes | No |
| **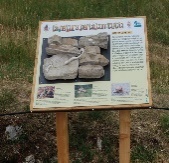** | **Trails** | Restoration | No | No |
| 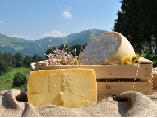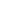 | **Local organic products** | Yes | No | No |
| 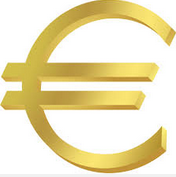 | **Entrance ticket** | 15 € | 18 € | 0 € |
| Which is your **preferred** scenario? | |  |  |  |
| Which one of the remaining is your **least preferred** scenario? | |  |  |  |

| **Card 2** | | **Scenario A** | **Scenario B** | **No local management** |
| --- | --- | --- | --- | --- |
| 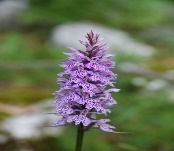 | **Meadow biodiversity** | Low | Medium | Low |
| 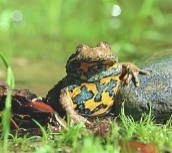 | **Yellow-bellied toad protection** | Yes | No | No |
| **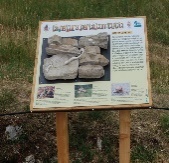** | **Trails** | Restoration and improvement | Restoration | No |
| 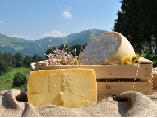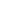 | **Local organic products** | No | Yes | No |
| 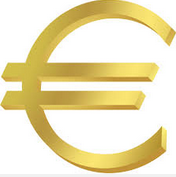 | **Entrance ticket** | 9 € | 6 € | 0 € |
| Which is your **preferred** scenario? | |  |  |  |
| Which one of the remaining is your **least preferred** scenario? | |  |  |  |

| **Card 3** | | **Scenario A** | **Scenario B** | **No local management** |
| --- | --- | --- | --- | --- |
| 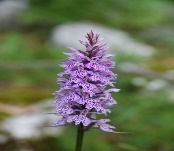 | **Meadow biodiversity** | Medium | High | Low |
| 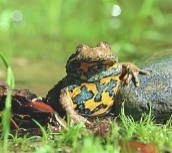 | **Yellow-bellied toad protection** | No | Yes | No |
| **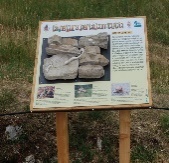** | **Trails** | Restoration and improvement | No | No |
| 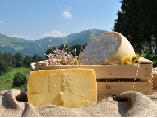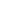 | **Local organic products** | No | Yes | No |
| 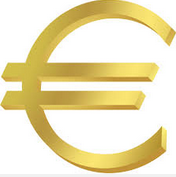 | **Entrance ticket** | 6 € | 9 € | 0 € |
| Which is your **preferred** scenario? | |  |  |  |
| Which one of the remaining is your **least preferred** scenario? | |  |  |  |

| **Card 4** | | **Scenario A** | **Scenario B** | **No local management** |
| --- | --- | --- | --- | --- |
| 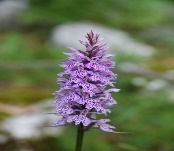 | **Meadow biodiversity** | High | Low | Low |
| 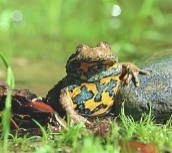 | **Yellow-bellied toad protection** | No | No | No |
| **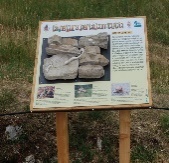** | **Trails** | No | Restoration | No |
| 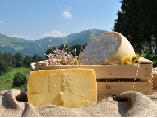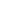 | **Local organic products** | No | Yes | No |
| 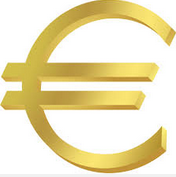 | **Entrance ticket** | 12 € | 15 € | 0 € |
| Which is your **preferred** scenario? | |  |  |  |
| Which one of the remaining is your **least preferred** scenario? | |  |  |  |

| **Card 5** | | **Scenario A** | **Scenario B** | **No local management** |
| --- | --- | --- | --- | --- |
| 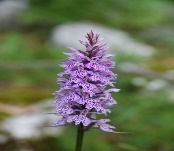 | **Meadow biodiversity** | High | Medium | Low |
| 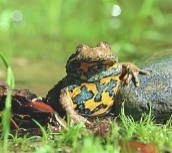 | **Yellow-bellied toad protection** | No | Yes | No |
| **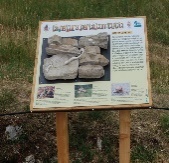** | **Trails** | Restoration | No | No |
| 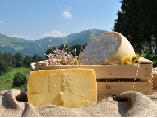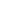 | **Local organic products** | Yes | No | No |
| 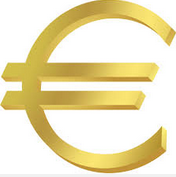 | **Entrance ticket** | 12 € | 6 € | 0 € |
| Which is your **preferred** scenario? | |  |  |  |
| Which one of the remaining is your **least preferred** scenario? | |  |  |  |

| **Card 6** | | **Scenario A** | **Scenario B** | **No local management** |
| --- | --- | --- | --- | --- |
| 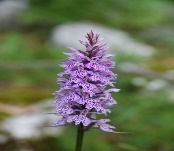 | **Meadow biodiversity** | Low | High | Low |
| 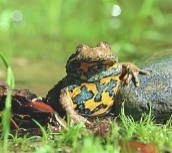 | **Yellow-bellied toad protection** | Yes | Yes | No |
| **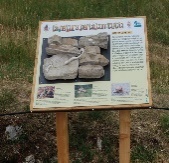** | **Trails** | No | Restoration and improvement | No |
| 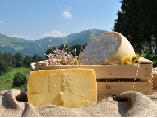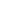 | **Local organic products** | Yes | No | No |
| 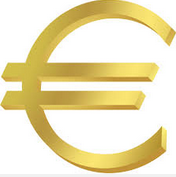 | **Entrance ticket** | 3 € | 15 € | 0 € |
| Which is your **preferred** scenario? | |  |  |  |
| Which one of the remaining is your **least preferred** scenario? | |  |  |  |

| **Card 7** | | **Scenario A** | **Scenario B** | **No local management** |
| --- | --- | --- | --- | --- |
| 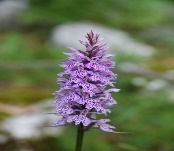 | **Meadow biodiversity** | Medium | High | Low |
| 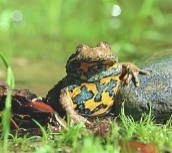 | **Yellow-bellied toad protection** | No | Yes | No |
| **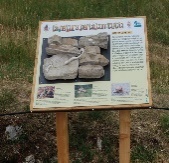** | **Trails** | Restoration and improvement | Restoration | No |
| 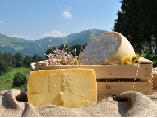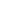 | **Local organic products** | Yes | No | No |
| 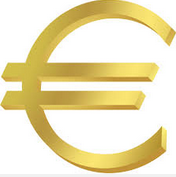 | **Entrance ticket** | 9 € | 12 € | 0 € |
| Which is your **preferred** scenario? | |  |  |  |
| Which one of the remaining is your **least preferred** scenario? | |  |  |  |

| **Card 8** | | **Scenario A** | **Scenario B** | **No local management** |
| --- | --- | --- | --- | --- |
| 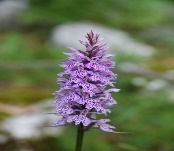 | **Meadow biodiversity** | Medium | Low | Low |
| 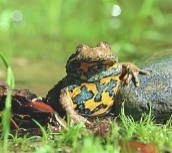 | **Yellow-bellied toad protection** | Yes | No | No |
| **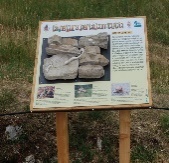** | **Trails** | No | No | No |
| 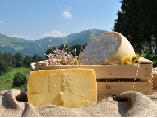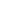 | **Local organic products** | No | Yes | No |
| 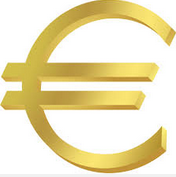 | **Entrance ticket** | 18 € | 12 € | 0 € |
| Which is your **preferred** scenario? | |  |  |  |
| Which one of the remaining is your **least preferred** scenario? | |  |  |  |

| **Card 9** | | **Scenario A** | **Scenario B** | **No local management** |
| --- | --- | --- | --- | --- |
| 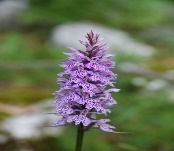 | **Meadow biodiversity** | High | Low | Low |
| 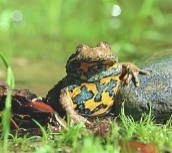 | **Yellow-bellied toad protection** | No | Yes | No |
| **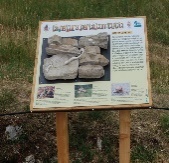** | **Trails** | No | Restoration and improvement | No |
| 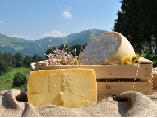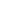 | **Local organic products** | No | Yes | No |
| 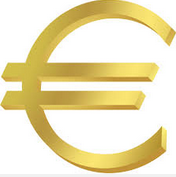 | **Entrance ticket** | 3 € | 18 € | 0 € |
| Which is your **preferred** scenario? | |  |  |  |
| Which one of the remaining is your **least preferred** scenario? | |  |  |  |

| **Card 10** | | **Scenario A** | **Scenario B** | **No local management** |
| --- | --- | --- | --- | --- |
| 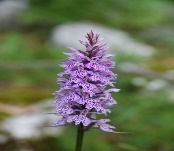 | **Meadow biodiversity** | Medium | Low | Low |
| 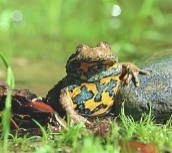 | **Yellow-bellied toad protection** | Yes | No | No |
| **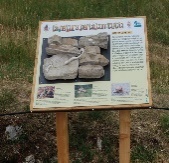** | **Trails** | Restoration | Restoration and improvement | No |
| 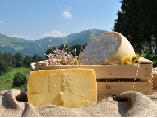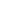 | **Local organic products** | Yes | No | No |
| 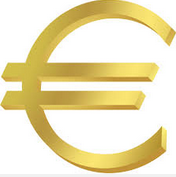 | **Entrance ticket** | 15 € | 3 € | 0 € |
| Which is your **preferred** scenario? | |  |  |  |
| Which one of the remaining is your **least preferred** scenario? | |  |  |  |

| **Card 11** | | **Scenario A** | **Scenario B** | **No local management** |
| --- | --- | --- | --- | --- |
| 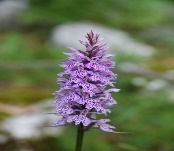 | **Meadow biodiversity** | Low | High | Low |
| 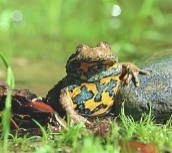 | **Yellow-bellied toad protection** | Yes | No | No |
| **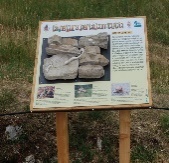** | **Trails** | Restoration | Restoration and improvement | No |
| 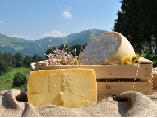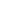 | **Local organic products** | No | Yes | No |
| 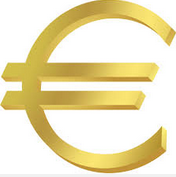 | **Entrance ticket** | 6 € | 9 € | 0 € |
| Which is your **preferred** scenario? | |  |  |  |
| Which one of the remaining is your **least preferred** scenario? | |  |  |  |

| **Card 12** | | **Scenario A** | **Scenario B** | **No local management** |
| --- | --- | --- | --- | --- |
| 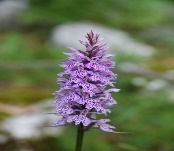 | **Meadow biodiversity** | High | Medium | Low |
| 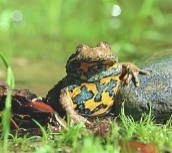 | **Yellow-bellied toad protection** | Yes | No | No |
| **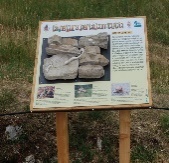** | **Trails** | Restoration and improvement | Restoration | No |
| 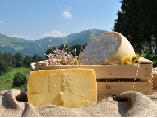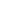 | **Local organic products** | Yes | No | No |
| 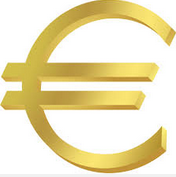 | **Entrance ticket** | 18 € | 3 € | 0 € |
| Which is your **preferred** scenario? | |  |  |  |
| Which one of the remaining is your **least preferred** scenario? | |  |  |  |

**Some concluding questions.**

**Please circle the number that best describes your opinion using a scale from 1 to 7.**

1. Concerning the daily weather conditions, are you:

Very 1 2 3 4 5 6 7 Very disappointed:________:________:________:________:________:________:________: satisfied

1. Please indicate how you feel right now about each emotion listed below.

|  | Not at all A lot | | | | | | |
| --- | --- | --- | --- | --- | --- | --- | --- |
|  | 1 | 2 | 3 | 4 | 5 | 6 | 7 |
| Happiness |  |  |  |  |  |  |  |
| Anxiety |  |  |  |  |  |  |  |
| Calmness |  |  |  |  |  |  |  |
| Irritability |  |  |  |  |  |  |  |
| Sadness |  |  |  |  |  |  |  |
| Boredom |  |  |  |  |  |  |  |

**Demographic questions:**

We remind you that this survey is anonymous.

The data collected will be used and disclosed only for statistical purposes in aggregate form.

1. **Do you identify yourself as:**

□ Male

□ Female

**2. What is your year of birth? ________________**

1. **What is your education?**

 Primary school

 Middle school

 Professional school

 High school

 Bachelor's degree

 Master's degree

 Ph.D. or similar

1. **What is your Zip code? _____________**
2. **What is your province of residence?______________**
3. **How would you describe your city of residence?**

 Big city (100.000 and more inhabitants)

 City (25.000-100.000 inhabitants)

 Town (2.500-25.000 inhabitants)

 Village (less than 2.500 inhabitants)

1. **Are you a member of any environmental association?**

 No

 Yes

1. **What was your after-tax income (in 2016)?**

|  Less than 9.999 € |  Between 40.000 € - 59.999 € |
| --- | --- |
|  Between 10.000 € - 19.999 € |  Between 60.000 € - 79.999 € |
|  Between 20.000 € - 29.999 € |  Between 80.000 € - 99.999 € |
|  Between 30.000 € - 39.999 € |  More than 100.000 € |

**INTERVIEW DATA:**

**QUESTIONNAIRE n^°^ ________**

**PLACE**

__________________________________________________________

**DATE**____/____/2017

**TIME________**

| Sunny  ❶ | Partly sunny  ❷ | Mostly cloudy  ❸ | Cloudy  ❹ | Rainy  ❺ |
| --- | --- | --- | --- | --- |

**WEATHER**

**WIND**

| Calm day  ❶ | Breezy  ❷ | Blustery  ❸ |  |  |
| --- | --- | --- | --- | --- |
